# Predictors of attitudes towards aging in elderly living in community care

**DOI:** 10.1186/s12877-024-04840-6

**Published:** 2024-03-18

**Authors:** Radka Bužgová, Radka Kozáková, Katka Bobčíková

**Affiliations:** Department of Nursing and Midwifery, Faculty of Medicine, University of Ostrava, Ostrava, Czech Republic

**Keywords:** Older people, Aging, Attitudes, Quality of life

## Abstract

**Background:**

Due to the aging of the population, the promotion of healthy aging is an important part of public health. Healthy aging of the population can be influenced by the attitudes of the elderly themselves towards old age and aging. The aim of this cross-sectional study was to find out the attitudes of older people living in a community environment toward old age and the predictors that influence these attitudes.

**Methods:**

The evaluation of attitudes towards old age using the WHO AAQ (Attitudes to Aging Questionnaire) questionnaire involved 1,174 elderly people living in the community. Age, sex, marital status, education, subjective health assessment, social support, depression (GDS-15), anxiety (GAI), sense of coherence (SOC-13) and self-esteem (RSES) were used to evaluate related factors.

**Results:**

As part of the exploratory factor analysis, a three-factor model (Psychosocial Loss, Physical Change, and Psychological Growth) was confirmed. The Cronbach alpha was found to be acceptable (α = 0.835). The predictors of better AAQ in the Psychological Loss domain were: subjective health, age, quality of life, self-esteem, sense of coherence, life satisfaction, anxiety, and social support; in the Physical Change domain: subjective health, quality of life, self-esteem, life satisfaction, cohabitation, and depression; and in the Psychological Growth domain: age, self-esteem, sense of coherence, life satisfaction, and social support.

**Conclusion:**

Preventive and policy measures should aim to increase the satisfaction and self-assessment of the elderly, which can help them evaluate the period of old age more positively. It is also important to create a positive perspective of ageing and elderly in society.

## Introduction

The Aging of the population is an unstoppable process and is becoming a global, society-wide problem. Promoting active aging and the health of the elderly is an important public health issue and is reflected in the health and social policies of individual countries. What older people and the elderly think and feel about old age and aging is an important psychological factor that contributes to health and personal well-being [1].

The period of old age brings with it a number of economic problems due to social changes including retirement or the loss of a partner [2]. With increasing age, health problems and associated limitations in normal daily activities also increase. These situations can negatively affect the attitudes of the elderly towards old age and aging and, consequently, their successful healthy aging [2, 3]. Healthy aging is perceived as an optimal level of functioning, active participation in society with meaningful participation, and acceptance of the normal aging process [4].

In addition to individual experiences, the influence of culture and social settings is an important factor in perceiving our own aging. Attitudes to aging vary across cultures, shaped by tradition, religion, and sociocultural beliefs [5, 6]. In some communities, there is tolerance and a positive attitude toward older people due to their experiences, memories, authority, and wisdom. While other more skeptical communities have a more negative attitude towards aging and perceive older people merely as ill. The image that the media creates of old age and aging can also have a significant impact on the perspective of older people themselves. The negative image of older people in advertising and media reinforces their sense of exclusion and takes away their sense of self-worth. These are factors that prevent older people’s diversity, potential, and competence from being recognized. People are social beings who need others to provide them with a sense of respect and self-esteem [7]. The influence of the social climate on the perception of the issues of old age and aging is very important, since it can influence self-assessment of aging.

What people think about older people and their own aging affects how they age themselves [8, 9] and how they behave [10–11]. Older people who have a positive view of aging have good psychological resources even in old age [12]. People with better attitudes toward aging show fewer negative effects, better health behavior, better health, lower mortality, better cognitive function, and lower risk of dementia compared to people with more negative attitudes toward their own aging [13–16]. Attitudes towards old age are also often associated with quality of life [2, 12, 17–21], life satisfaction, successful aging [2,11,] and fragility [22]. Some research confirms that negative attitudes to old age are more common in people with poor physical health [23–25], more comorbidities [26], and lower functional level [26], or in those with depression [27]. Yamada et al. [28] suggest that the negative impact of comorbidity on quality of life can be mitigated by promoting a positive perception of aging in older people.

Social support can play an important role in the perception of one’s own aging. Social support includes instrumental support (offering a helping hand), emotional support (making older people feel loved), and informational support (providing older people with beneficial information) [29]. All of these components are important in promoting a positive view of old age.

## Methods

### Aim

The aim of this cross-sectional study was to find out the attitudes of older people living in a community environment toward old age and the predictors that influence these attitudes. We assumed that predictors of attitudes toward old age might be demographic factors, anxiety, depression, subjective assessment of quality of life, self-esteem, sense of coherence, level of social perceived support, and subjective assessment of health. The cross-sectional study also evaluated the psychometric properties of the Czech version of the Attitudes to Aging Questionnaire (AAQ).

### Study design and sample

A total of 1,174 older people from the Moravian-Silesian Region who live in a home environment participated in a cross-sectional study. According to data from the Czech Statistical Office in 2021, there are approximately 236,000 people over 65 years of age living in the Moravian-Silesian region. Our group comprised 0.5% of these seniors. The criterion for inclusion in the research group was age 60 or older, cognitively intact (no diagnosed dementia, ability to sign an informed consent form). Older people were approached in all districts of the Moravian-Silesian Region through more than ten organizations (e.g., senior clubs, community centers), through libraries, and also through the Center for Prevention and Promotion of Healthy Aging of the Medical Faculty, University of Ostrava). The questionnaires were distributed to older people in both printed and electronic format from September 2021 to December 2022. The paper form of the questionnaire was given to the older people who visited the centers in an envelope by a research assistant. The questionnaire was filled in at home, then returned and passed on to a center’s worker or research assistant. A link to the electronic version of the questionnaire was sent to older people by email. Each participant had access to fill out only once from the link. The electronic version of the questionnaire was sent to older people who chose this format. The printed and electronic versions of the questionnaire contained the same instructions for filling in. The individual questionnaires were submitted in the same order in both versions.

## Measures

The following set of questionnaires was used to collect the data.

### Assessment of attitudes to aging

- Attitudes to Aging Questionnaire– AAQ [30, 31]. The 24 items on the AAQ scale are scored on a five-point Likert scale (1 = strongly disagree, 5 = strongly agree). It consists of three broad dimensions of aging: 1. Physical change (eight items that include health, dynamics, vitality, and exercising), 2. Psychological growth (eight elements, which reflect explicit gains in relation to self and others; a positive focus on aging, orientation of life, connection with ‘wisdom’ and ‘fruits of life,’ coping, acceptance, and communication with younger generations), 3. Psychosocial loss (eight items, in which old age is described primarily as a negative experience, including loss, deficiency, exclusion, loss of independence, depression, and loneliness). More positive attitudes to ageing are indicated by higher scores.

### Evaluation of selected factors and predictors

- *The Geriatric Depression Scale– GDS-15* [32]. The GDS may be used with healthy, medically ill, and mild to moderately cognitively impaired older people (MMSE-score above 18 points), [33]. A Short Form GDS consisting of 15 questions was developed in 1986. The GDS-15 score range is 0 to 15 points, with higher scores indicating greater depressive symptoms. Scores of 0–4 are considered normal; 5–8 indicate mild depression; 9–11 indicate moderate depression; and 12–15 indicate severe depression.
- *Geriatric Anxiety Inventory* GAI [34]. The GAI scale consists of 20 items (‘Agree/disagree’) designed to assess typical common anxiety symptoms. The sum of these ratings is a measure of general anxiety symptoms (ranged from 0 to 20), with higher scores indicating greater anxiety.
- *Older People Quality of life brief– OPQOL_brief* questionnaire [35]. The scale consists of 13 statements. Respondents indicate to what extent they agree with each statement by selecting one of five possible options (“strongly disagree”, “disagree”, “neither agree or disagree”, “agree” and “strongly agree”). Items scores are totaled to provide a total OPQoL-brief score ranging from 13 to 65, with higher scores indicating better QoL [35].
- *The Sense of Coherence Scale - SOC-13* [36]. The short form of the SOC scale consists of 13 items that comprise three components: comprehensibility– SOC_C (5 items), manageability– SOC_MA (four items), and meaningfulness– SOC_ME (four items). Respondents indicate agreement or disagreement on a seven-category semantic differential scale with two anchoring responses tailored to the content of each item. The total score can range from 13 to 91, with a higher score indicating a higher SOC. The sub-score ranges as follows: SOC_C from 5 to 35; SOC_MA and SOC_ME from 4 to 28.
- *Life Satisfaction Index for the Third Age– Short Form - LSITA-SF* [37] (Barrett, Murk 2009). LSITA-SF consists of 12 items and uses a six-point Likert scale: Strongly disagree (6); disagree (5); disagree somewhat (4); agree somewhat (4); agree (2); and strongly agree (1). The scores for the twelve items are totaled to establish the Life Satisfaction score (12–72 points). Higher scores indicate higher life satisfaction.
- *Rosenberg Self-Esteem Scale– RSES* [38] consists of ten items rated on a four-point Likert type scale from Strongly Agree (SA) to Strongly Disagree (SD). A score of 15–30 = normal self-esteem; while a score of less than 15 = low self-esteem.

Age, sex, marital status, co-habitation, employment, and social support were the social factors assessed. Social support was evaluated with one item: ‘Do you have the impression that you have people close to you who will give you help and support if you need it?’ on a scale of 1 (yes, always) to 10 (no, never). Next, a Social Support 6 (SS-6) questionnaire made up of six items: *“Do you have someone who will take care of you, no matter what?”; “Will they calm you down if you feel unnerved?”; “Will they really help you if you feel devastated?”; “Do they accept you as you are?”; “Will they dispel your fears and anxieties when you are stressed?”; “Will they help you with your day-to-day business?”* The scores on the SS-6 are 0–6, with higher scores indicating greater social support.

Subjective health assessments were collected through one item: *“How would you evaluate your health?”* with possible answers: (1) bad, I need assistance; (2) quite bad, my health significantly limits me in everyday activities; (3) quite good, my health does not significantly limit me in daily activities; (4) good, I feel fully-healthy, and do not perceive limitations in performance and daily activities.

### Ethical consideration

The study was conducted in accordance with the provisions of the Declaration of Helsinki and was approved by the ethics committees of the University of Ostrava, Faculty of Medicine (no. 14/2020). All subjects gave their informed consent to inclusion before participating in the study.

### Statistical analysis

The SPSS statistical program, v. 24.0, was used for data analysis. First, we tested the psychometric properties of the AAQ scale. We performed an exploratory factor analysis, a method of determining the major components with Varimax rotation, to help us better understand the factor structure of the AAQ questionnaire. Before the factor analysis was performed, the suitability of using the KMO (Kaiser-Meyer-Olkin measure) and the Bartlett sphericity test was verified. The model was tested as a three-factor model [31]. Internal consistency was determined by Cronbach’s alpha coefficient (α). Furthermore, we assessed the correlation of the individual items and the given domain (item-total correlation).

In addition, data were evaluated using descriptive statistics (absolute and relative frequency, mean, standard deviation). Differences between groups were evaluated using the Kruskal-Wallis test and the independent Wilcoxon test. The correlation between the selected parameters was determined using the Spearman correlation coefficient. Nonparametric tests were used due to abnormal data distribution (Shapiro–Wilk’s test and Kolmogorov-Smirnov test, *p* < 0.001). The strength of the relationship according to the correlation coefficient r was evaluated as follows: 0 - zero; ±0.1–0.3 weak; ±0.4–0.6 moderate; ±0.7–0.9 strong; ±1 perfect [39].

Multivariate regression analysis was performed using the enter method, employing all variables that showed significant association (*p* < 0.05) in the primary analysis. The quality of the model was evaluated by the coefficient of determination (R^2^).

## Results

The sample consisted of 1,174 elderly people. The average age of the entire sample was 72.28 years (s = 6.15; min/max = 60/96 years). The majority (71%) were women and people no longer working (84%). Just over a quarter of older people (26%, *n* = 299) had experienced the death of a close person in the last year. The sociodemographic characteristics of the sample are shown in Table 1. Older people were the most likely to subjectively rate their health as good (68%). A total of 11% of the older people surveyed rated their health fair or poor (see Table 1). A total of 1,073 (91.4%) older people were treated for some chronic diseases on a regular basis. On average, an older adult was treated for 2.4 (SD = 1.7) diseases. These were chronic diseases: cardiovascular (61.6%), oncological (6.7%), diabetic (17.8%), endocrinology (17.1%), respiratory (13.7%), gynecological (3.6%), urological (15.5%), sensory (26.3%), musculoskeletal (44.5%), neurological (7.6%), mental health problem (4.3%).

**Table 1:** Sociodemographic and subjective health characteristics of the sample (*n* = 1174)

Age	n	%	Physical and mental health	n	%
60–74 years	840	71.6	**Subjective Physical Health Assessment**		
≥ 75 years	334	28.4	Very good	253	21.6
**Gender**			Good	794	67.6
Man	343	29.2	Fair	107	9.1
Women	831	70.8	Poor	20	1.7
**Marital status**			**BMI**		
Single	45	3.8	< 18.5	11	0.9
Married	564	48.0	18.5–25.0	323	27.5
Divorced	198	16.9	25.1–30.0	505	43.0
Widow	367	31.3	> 30.0	335	28.6
**Employment**			**GDS-15**		
Full time	53	4.5	0–5	992	84.5
Part-time job	138	11.7	6–10	139	11.8
None	983	83.7	> 10	43	3.7
**Living with**			**GAI**		
No-one	485	41.3	0–8	916	78.0
Spouse	568	48.4	> 8	258	22.0
Children	72	6.1	**Scales**	**mean**	**s**
Another	49	4.2	GDS-15	3.0	3.0
**Religion**			GAI	4.7	5.2
Yes, Christian	331	28.2	RSES	19.5	3.4
Yes	306	26.1	OPQOL_brief	54.6	6.6
No	537	45.7	LSITA	48.9	8.2
**Social support (SS-6)**			SOC-13	62.2	9.9
0–2	79	6.7	SOC_C	22.2	4.8
3–4	149	12.7	SOC_ME	21.7	3.7
5–6	946	80.6	SOC_MA	18.4	3.8

BMI– Body max index, GDS-15– Geriatric Depression Scale, GAI– Geriatric Anxiety Inventory, RSES - Rosenberg Self-Esteem Scale, OPQoL brief - Older People Quality of life brief, LSITA-SF - Life Satisfaction Index for the Third Age– Short Form, SOC-13– Sense of Coherence scale, SOC_C- comprehensibility, SOC_ME - meaningfulness, SOC_MA - manageability, SS-6– Social Support-6

First, we performed an exploratory factor analysis. The Kaiser-Meyer-Olkin measure was found to be adequate (0.871), exceeding the recommended minimum value of 0.60. The compliance parameters were also found to be according to Bartlett’s sphericity test (A: chí2 = 6982.728; Df = 276; *p* < 0.001), therefore factor analysis could be performed. As part of the exploratory factor analysis, a three-factor model was confirmed, dividing the items into three domains (see Table 2). Loading factors were found for all items in the given domain greater than 0.4, except item no. 10– *“I am more accepting of myself as I have grown older”* from the Psychological Growth domain. A low correlation value between items (*r* = 0.166) was found in this item. The Cronbach alpha was found to be acceptable (α ˃ 0.7) in all three domains, even in the overall rating of all items (α = 0.835). Adequate reliability was also found for the other scales used (see Table 3).

**Table 2:** Results of exploratory factor analysis

	Mean + SD	Item-total cor	Psychosocial Loss	Physical Change	Psychological Growth
3. Old age is a time of loneliness.	3.5 (0.9)	0.393	0.671		
6. Old age is a depressing time of life.	3.6 (0.9)	0.471	0.587		
9. I find it more difficult to talk about my feelings as I get older.	3.2 (1.0)	0.285	0.625		
12. I see old age mainly as a time of loss.	3.2 (1.0)	0.312	0.580		
15. I am losing my physical independence as I get older.	3.0 (1.0)	0.355	0.494		
17. As I get older, it becomes more difficult to make new friends.	3.0 (1.1)	0.333	0.610		
20. I don’t feel involved in society now that I am older.	3.3 (1.0)	0.451	0.722		
22. I feel excluded from things because of my age.	3.1 (1.0)	0.490	0.671		
7. It is important to take exercise at any age.	4.0 (0.8)	0.311		0.425	
8. Growing older has been easier than I thought.	3.2 (1.0)	0.524		0.510	
11. I don’t feel old.	3.5 (0.9)	0.526		0.623	
13. My identity is not defined by my age.	3.8 (0.8)	0.332		0.492	
14. I have more energy now than I expected for my age.	3.3 (0.9)	0.519		0.698	
16. Problems with my physical health do not hold me back from doing what I want to.	3.4 (0.9)	0.450		0.626	
23. My health is better than I expected for my age.	3.3 (0.9)	0.467		0.678	
24. I keep myself as fit and active as possible by exercising.	3.6 (0.9)	0.422		0.599	
1. As people get older, they are better able to cope with life.	3.3 (0.8)	0.373			0.595
2. It is a privilege to grow old.	3.4 (0.9)	0.242			0.522
4. Wisdom comes with age.	3.2 (0.9)	0.189			0.639
5. There are many pleasant things about growing older.	3.2 (0.8)	0.501			0.514
10. I am more accepting of myself as I have grown older.	3.5 (0.8)	0.162			0.389
18. It is very important to pass on the benefits of my experience to younger people.	3.6 (0.8)	0.304			0.642
19. I believe my life has made a difference.	4.0 (0.6)	0.504			0.489
21. I want to give a good example to younger people.	3.8 (0.7)	0.310			0.566
Explained Variance			22.3%	11.6%	6.4%
**Cronbach alpha**		0.835	0.793	0.779	0.714
**Total score (mean)**	82.05		25.97	28.19	27.89
**Skewness**			-0.128	-0.491	-0.698
**Kurtosis**			-0.341	0.480	2.538

**Table 3:** Reliability of used scales (Cronbach’s alpha)

Scales	Cronbach’s alpha	Scales	Cronbach’s alpha
GDS-15	0.811	SOC-13	0.758
GAI	0.919	RSES	0.799
OPQOL_brief	0.913	LSITA-SF	0.868
SS-6	0.800		

GDS-15– Geriatric Depression Scale, GAI– Geriatric Anxiety Inventory, OPQOL_brief - Older People Quality of life brief, SS– social support 6, SOC-13– Sense of Coherence scale, RSES - Rosenberg Self-Esteem Scale, LSITA-SF - Life Satisfaction Index for the Third Age– Short Form

Subsequently, we evaluated the association between the AAQ domains and the selected factors using correlation analysis. Age was not related to the Psychological Growth domain. In this domain, only a weak degree of correlation was found with other items. Table 4 shows in bold the correlation coefficient values that indicate a moderate degree of correlation (*r* = 0.4–0.6). In the Psychosocial Loss domain, there was a moderate degree of correlation with quality of life (positive), anxiety (negative), depression (negative), self-esteem (positive), life satisfaction (positive), and a sense of coherence (positive). In the domain of Physical Change, there were correlations with subjective health (positive), quality of life (positive), depression (negative), and life satisfaction (positive). For other variables, the correlations were also statistically significant, but weak.

**Table 4:** Correlation analysis of AAQ domains with selected factors

	Psychosocial Loss		Physical Change		Psychological Growth	
	r	*p*	r	*p*	r	*p*
Subjective health	0.338	0.000	**0.441**	0.000	0.091	0.002
Age	-0.203	0.000	-0.078	0.007	0.004	0.887
Total QOL	**0.448**	0.000	**0.433**	0.000	0.207	0.000
OPQOL_brief	**0.508**	0.000	**0.519**	0.000	0.283	0.000
GDS-15	**-0.516**	0.000	**-0.465**	0.000	-0.273	0.000
GAI	**-0.423**	0.000	-0.305	0.000	-0.224	0.000
RSES	**0.406**	0.000	0.359	0.000	0.235	0.000
LSITA-SF	**0.580**	0.000	**0.497**	0.000	0.378	0.000
SOC-13	**0.431**	0.000	0.361	0.000	0.283	0.000
SOC_C	0.338	0.000	0.265	0.000	0.203	0.000
SOC_ME	0.390	0.000	0.380	0.000	0.308	0.000
SOC_MA	0.324	0.000	0.230	0.000	0.181	0.000
Social support	-0.262	0.000	-0.163	0.000	-0.188	0.000
SS-6	0.271	0.000	0.176	0.000	0.205	0.000

QOL– quality of life, OPQOL_brief - Older People Quality of life brief, GDS-15– Geriatric Depression Scale, GAI– Geriatric Anxiety Inventory, RSES - Rosenberg Self-Esteem Scale, LSITA-SF - Life Satisfaction Index for the Third Age– Short Form, SOC-13– Sense of Coherence scale, SOC_C- comprehensibility, SOC_ME - meaningfulness, SOC_MA - manageability, SS– social support 6, bold - a moderate degree of correlation

We also evaluated differences in age attitudes by sex, cohabitation, employment, and marital status (see Table 5). Older people living with a partner or other person in the same household reported more positive attitudes to old age in the area of Psychosocial Loss (*p* = 0.044). Furthermore, women reported more positive attitudes to old age than men in the area of Physical Change (*p* = 0.007). Older people who worked full-time or part-time reported more positive attitudes to age in all three domains. According to family status, a statistically significant difference (*p* = 0.038) was found only in the domain of Psychosocial Loss. Widowers and widows, in particular, reported worse attitudes toward old age and aging.

**Table 5:** Comparison of attitudes towards old age according to selected sociodemographic factors

	Psychosocial Loss		Physical Change		Psychological Growth	
	Mean (SD)	*p*	Mean (SD)	*p*	Mean (SD)	*p*
**Gender**						
Man (*n* = 343)	25.8 (5.1)	0.308	27.6 (4.5)	**0.007**	27.9 (3.4)	0.954
Women (*n* = 831)	26.0 (4.9)		28.4 (4.3)		27.9 (3.8)	
**Living with**						
Alone (*n* = 485)	25.5 (5.2)	**0.044**	28.3 (4.5)	0.244	27.7 (3.9)	0.089
With (*n* = 689)	26.3 (4.8)		28.1 (4.3)		28.0 (3.5)	
**Working**						
No (*n* = 983)	25.6 (4.9)	**0.000**	28.0 (4.4)	**0.001**	27.7 (3.7)	**0.007**
Yes (*n* = 191)	27.6 (4.9)		29.4 (3.9)		28.6 (3.7)	
**Marital status**						
Single (*n* = 45)	26.2 (5.3)	**0.038**	28.5 (4.2)	0.755	27.6 (3.3)	0.942
Married (*n* = 564)	26.3 (4.7)		28.1 (4.2)		27.8 (3.4)	
Divorced (*n* = 198)	26.7 (5.1)		28.6 (4.3)		27.9 (3.6)	
Widow (*n* = 367)	25.2 (5.3)		27.9 (4.7)		27.9 (4.1)	

Table 6 shows predictors of attitudes towards aging (domains: Psychosocial Loss, Physical Change, and Psychological Growth). All three models were confirmed to be statistically significant. The predictors explained 44.5% of the variation in the Psychosocial Loss domain, 44.1% of the variation in the Physical Change domain, and only 15.4% of the variation in the Psychological Growth domain. The predictors of better AAQ in the Psychological Loss domain were: subjective health, age, quality of life, self-esteem, sense of coherence, life satisfaction, anxiety, and social support. The predictors of better AAQ in the Physical change domain were: subjective health, quality of life, self-esteem, life satisfaction, cohabitation, and depression The predictors of better AAQ in the Psychological growth domain were: age, self-esteem, sense of coherence, life satisfaction, and social support.

**Table 6:** Multiple regression analysis with the selected factors as independent variables and the AAQ domains as dependent variables

Selected factors	Psychosocial Loss R^2^ = 0.445, F = 116,611, df = 8, *p* = 0.000		Physical Change R^2^ = 0.411, F = 137,349, df = 6, *p* = 0.000		Psychological Growth R^2^ = 0.154, F = 36,614, df = 6, *p* = 0.000	
	β	*p*	β	*p*	β	*p*
Subjective health	0.097	0.000	0.231	0.000	---	---
Age	-0.103	0.000	---	---	0.061	0.027
Total QOL	0.164	0.000	0.220	0.000	---	---
RSES	0.066	0.016	0.115	0.000	0.095	0.005
SOC	0.059	0.042	---	---	0.087	0.010
LSITA	0.304	0.000	0.172	0.000	0.258	0.000
GAI	-0.135	0.000	---	---	---	---
Social support	0.070	0.003	---	---	0.067	0.018
Living with	---	---	-0.057	0.011	---	---
GDS	---	---	-0.125	0.000	---	---

QOL– quality of life, RSES - Rosenberg Self-Esteem Scale, SOC– Sense of Coherence, LSITA - Life Satisfaction Index for the Third Age– Short Form, GAI– Geriatric Anxiety Inventory, GDS– Geriatric Depression Scale

## Discussion

Evaluating older people’ attitudes toward old age is important for evaluating the effectiveness of interventions aimed at supporting old age and aging. The Attitude to Aging Questionnaire (AAQ) was developed to assess perceptions of the aging process among older people [31]. The AAQ scale has been used with a number of population groups across several languages, including English, Spanish, Czech, Norwegian, German, Danish, French, Hungarian, Hebrew/Arabic, Japanese, Swedish, Portuguese, Turkish, Lithuanian, and Chinese [1]. The advantage of using an international questionnaire is a possible comparison of data between individual countries. Burton et al. [1], in their systematic overview, consider the use of this questionnaire to be suitable for research and practice but recommend further investigation of the psychometric properties of the AAQ scale. For this reason, we also evaluated the psychometric properties of the Czech version of the AAQ scale as part of our cross-sectional study. The original three-factor model [31], which divides items into three domains, was confirmed by factor analysis: (1) Physical Functioning, (2) Psychological Growth, (3) Psychosocial Losses. The three-factor model was also confirmed in the Spanish [40], Iranian [41], Portuguese [42], and Brazilian versions [27]. As part of our investigation, we found satisfactory reliability. The Cronbach alpha of the entire questionnaire was α = 0.84. Laidlaw et al. [31] reported a similar Cronbach alpha value when testing the original version of the questionnaire (α = 0.86), as did Pedro de Lima et al. [42] when testing the Portuguese version (α = 0.84). Compliant reliability was also found in all AAQ domains. Some psychometric studies of the AAQ report a value of less than 0.7 in one domain [31, 42].

Furthermore, we evaluated the link between attitudes to old age and aging in individual domains and selected factors. In particular, links between better attitudes towards old age and better quality of life, life satisfaction, self-esteem, sense of coherence, subjective health assessment, social support, and lower anxiety and depression were demonstrated in our research. Thus, these are important factories that are interrelated and can significantly influence the healthy aging of the population. Lucas-Carrasco et al. [40] point out the link between attitudes to old age and depression levels and the number of physical comorbidities. Buckinx et al. [43] state that poorer subjective health assessment and a higher number of comorbidities occur in frail seniors, who tend to demonstrate worse attitudes to old age than the non-frail.

Better attitudes towards old age and aging in all domains were found in people who were still working part-time or full-time. This is one of the key findings of our research. Maintaining employment for older people for as long as possible can help them perceive the period of old age and aging more positively. Furthermore, older people who lived with someone in the same household reported better attitudes in the Psychosocial Loss domain compared to those who lived alone. Widows also reported worse attitudes in this domain. These findings indicate the need of supporting the maintenance of employment in old age and the need of supporting interventions aimed at the social support of the elderly. By gender, we found a difference only in the Physical Change domain ratings, with women reporting better attitudes in this domain than men. Some studies have shown a link between attitudes towards old age and education [40]. Differences in attitudes towards old age by marital status, education, employment, living in the home, subjective health, and quality of life are reported in Cadmus et al. [44]. The authors point to age as a significant factor. In our research, only a weak degree of correlation was found between age and attitudes towards old age. Age was found to be a predictor in the domain of Psychosocial Loss. Similarly, Kisvetrová et al. [45] found that age is a predictor of AAQ, in addition to gender and physical ability.

The main objective of our research was to identify the predictors of older peoples’ attitudes toward old age and aging, which differed slightly from one domain to another. The common predictors for all three domains were life satisfaction and self-esteem. Preventive and policy measures should aim to increase the satisfaction and self-assessment of older people, which can help them evaluate the period of old age more positively. Meaningfulness of life is an important motivating element, encouraging us to make sense of the outside world. A person with a strong sense of cohesion can better cope with a range of difficult life situations [46]. Kornadt et al. [47] state that personality is longitudinally related to changes in attitudes toward our own aging: lower neuroticism, higher conscientiousness, and greater openness predicted more positive attitudes, whereas the effect of extraversion varied with time. Self-compassion can also be an internal resource for promoting healthy attitudes towards aging, and thus for promoting healthy aging in society as a whole [48]. Miche et al. [49] demonstrated that attitudes toward aging may be more amenable to change in midlife than in later life. Early interventions must be ensured to promote healthy aging and a healthy view of old age in middle-aged and younger people. It is important to create a positive perspective of ageing and elderly in all society because as we mentioned, the image that the media creates of old age and ageing can also have a significant impact on the perspective of older people themselves.

Jang and Kim [2] cite depression as a predictor of worse attitudes toward old age. In our research, depression as a statistically significant predictor of attitudes towards old age was confirmed only in the domain of Physical Change. Older people with depression should be actively sought out in community care and treated in a timely manner. Cadmus et al. [44] showed, through regression analysis, that a better attitude towards old age is found in the educated, employed, and those with good self-assessment of health. In our research, subjective health assessment and overall quality of life were predictors of attitudes in two domains: namely Psychosocial Loss and Physical Changes. Korkmaz Aslan [11], on the other hand, investigated whether attitudes toward old age are predictors of quality of life by multiple regression analysis. In their research, they found that Psychosocial Loss, Physical Change, and Psychological Growth were statistically significant predictors of quality of life among older people living in the community in Turkey.

Social support was another factor that influenced the situation. Social support was a significant predictor in the area of Psychological Loss and Psychological Growth. Liu et al. [50] notes the importance of social support for the evaluation of attitudes toward old age. Support for social relationships and a program aimed at reducing loneliness should be part of the measures supporting healthy aging. More research is needed to determine whether targeted psychosocial interventions aimed at promoting self-esteem, sense of coherence, treating depression and anxiety, improving or maintaining health, and promoting employment can improve older peoples’ view of their own age and aging.

### The limits of the study and future research

A main limitation of our study is the sampling methods, specifically the use of a convenience sampling strategy, that is, nonrandom sampling. Therefore, not all members of the population had the same probability of being selected. Stratton [51] states that conventional sampling can be used for population and clinical research. The analysis of the results of the convenience sample can only be applied to the study participant group. Importantly, associations and effects found with a convenience sample cannot be generalised to a target population. On the other hand, convenience sampling is cheaper, quicker, and simpler than other forms of sampling.

Random sampling in a whole group of the older adult population is very problematic. This is a very heterogeneous group of the population. In our investigation, we focused on a group of older people who attend the mentioned centers. The suggested recommendations are directed to this group. We consider it important to promote healthy ageing among this group of older people and thereby increase the likelihood of prolonging their active life in society. We deliberately did not include people with cognitive impairment in the research file. The questionnaire used is not intended for these people.

For further research, we recommend also targeting older people who live in a home environment and do not attend community centers or are cognitively impaired, e.g., through general practitioners. For this group, propose specific recommendations.

Another limit of research can be the use of both electronic and printed versions of the questionnaire. For both versions, we decided to allow older people to choose which format to fill in, thus increasing the likelihood of returning the questionnaires. Both the electronic and printed versions provided the same information to fill out the questionnaire, as well as the same order for the individual questionnaires.

## Conclusion

The promotion of the subjective perception of one’s own aging and old age can be an important salutary factor, playing an important part in the healthy aging process and helping to prevent the deterioration of the mental and physical health of the elderly. Targeted psychosocial interventions should be applied in a timely manner as part of health social policy measures, i.e., in late adulthood or early old age.

## Data Availability

The datasets used and/or analysed during the current study are available from the corresponding author on reasonable request.
